# Synthesis and *in vitro* anti-proliferative activity of some novel isatins conjugated with quinazoline/phthalazine hydrazines against triple-negative breast cancer MDA-MB-231 cells as apoptosis-inducing agents

**DOI:** 10.1080/14756366.2017.1279155

**Published:** 2017-02-08

**Authors:** Wagdy M. Eldehna, Hadia Almahli, Ghada H. Al-Ansary, Hazem A. Ghabbour, Mohamed H. Aly, Omnia E. Ismael, Abdullah Al-Dhfyan, Hatem A. Abdel-Aziz

**Affiliations:** a Department of Pharmaceutical Chemistry, Faculty of Pharmacy, Egyptian Russian University, Badr City, Cairo, Egypt;; b Department of Chemistry, Faculty of Pharmacy, University of Oxford, Oxford, UK;; c Department of Pharmaceutical Chemistry, Faculty of Pharmacy, Ain Shams University, Cairo, Abbassia, Egypt;; d Department of Pharmaceutical Chemistry, College of Pharmacy, King Saud University, Riyadh, Saudi Arabia;; e Department of Pharmacology and Toxicology, Faculty of Pharmacy, British University in Egypt, Cairo, Egypt;; f Department of Biology, The American University in Cairo, New Cairo, Egypt;; g Department of Biochemistry, Faculty of Pharmacy, Egyptian Russian University, Badr City, Cairo, Egypt;; h Stem Cell & Tissue Re-Engineering Program, Research Center, King Faisal Specialized Hospital & Research Center, Riyadh, Saudi Arabia;; i Department of Applied Organic Chemistry, National Research Center, Dokki, Giza, Egypt

**Keywords:** Isatin, quinazoline, phthalazine, synthesis, triple-negative breast cancer

## Abstract

Treatment of patients with triple-negative breast cancer (TNBC) is challenging due to the absence of well- defined molecular targets and the heterogeneity of such disease. In our endeavor to develop potent isatin-based anti-proliferative agents, we utilized the hybrid-pharmacophore approach to synthesize three series of novel isatin-based hybrids **5a**–**h**, **10a**–**h** and **13a**–**c**, with the prime goal of developing potent anti-proliferative agents toward TNBC MDA-MB-231 cell line. In particular, compounds **5e** and **10g** were the most active hybrids against MDA-MB-231 cells (IC_50_ = 12.35 ± 0.12 and 12.00 ± 0.13 μM), with 2.37- and 2.44-fold increased activity than 5-fluorouracil (5-FU) (IC_50_ = 29.38 ± 1.24 μM). Compounds **5e** and **10g** induced the intrinsic apoptotic mitochondrial pathway in MDA-MB-231; evidenced by the reduced expression of the anti-apoptotic protein Bcl-2, the enhanced expression of the pro-apoptotic protein Bax and the up-regulated active caspase-9 and caspase-3 levels. Furthermore, **10g** showed significant increase in the percent of annexin V-FITC positive apoptotic cells from 3.88 to 31.21% (8.4 folds compared to control).

## Introduction

Heading the list of the critical health problems for females, breast cancer continues to be the major life-threatening health issue. According to the recent records, an estimated 1.7 million women are expected to be diagnosed with breast cancer by 2020 with a make-up of 26% increase from current recorded levels^1^^,^^2^. Despite the huge and continuous efforts of the scientific community, no tangible improvements were recorded in the last decade. With the development of resistance toward the available anticancer drugs, the mission becomes even more challenging and the efficacious therapy remains questionable^3^^,^^4^.

Targeting breast cancer with tailored drugs depends on the fact of immunohistochemical expression of estrogen receptors (ERs), progesterone receptors (PRs) and human epidermal growth factor receptors-2 (HER-2). Being devoid of the three aforementioned targetable proteins, triple-negative breast cancer (TNBC) stands even as an unbeatable challenge^5^. Besides their well-known resistance to the current chemotherapy, TNBC is an aggressive phenotype of cancer with high recurrence risk, poor prognosis, reduced survival and distant metastasis that it may reach the lungs and even the central nervous system (CNS)^5^^,^^6^. Accordingly, patients largely receive systemic chemotherapy that puts them under higher risk of suffering the devastating side effects. This urgent necessity motivates medicinal chemists to search for alternative treatments targeting apoptotic machinery as a novel approach for TNBC therapy.

Targeting critical regulators of apoptosis with the goal of inducing apoptosis in cancer cells still stands as an attractive and successful strategy to drug discovery and development of new anti-cancer agents^7–11^. On April 11, 2016, the U.S. food and drug administration (FDA) approved Venetoclax (Venclexta^®^) for the treatment of patients with chronic lymphocytic leukemia (CLL) whose tumors have a specific genetic alteration. Venclexta is the first FDA-approved drug that targets the BcL-2 protein, an anti-apoptotic regulatory protein^12^.

Molecular hybridization is a contemporary concept in drug design and development gaining momentum worldwide. There are two main ways that can be explored to design and construct affordable and efficient hybrid molecules. The first method merges haptophoric moieties of different drugs and the second technique combines two or more entire drugs together using a linker chain^13^^,^^14^. This strategy affords novel molecule hybrids with improved affinity and efficacy when compared to the parent drugs, modified selectivity profile, different and/or dual modes of action and reduced undesired side effects. Thus, hybrid molecules emerged as magic bullets that trigger two or more cytocidal pharmacological mechanisms of action acting in synergy to inhibit cancer tumor growth^15–17^.

**Figure 1.**
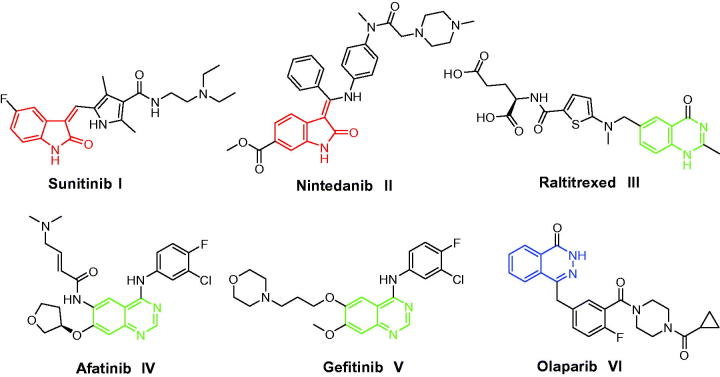
Structures of some isatin-, quinazoline- and phthalazine-based **I–VI** approved anticancer drugs.

Pertaining to its wide presence endogenously in both human and other mammalian tissues and fluids besides its prevalence in many naturally occurring compounds, notably alkaloids, fungal metabolites and marine natural products, isatin (1*H*-indole-2,3-dione) as a privileged scaffold is endowed with excellent anticancer profile^18^. Thus, medicinal chemists embarked on exploring diverse isatin derivatives comprehending their potential to create novel bioactive compounds. By 2006, the FDA approval of Sunitinib (Sutent^®^) **I** (Figure 1) for the treatment of advanced renal carcinoma and gastrointestinal stromal tumors furnished the path for the creation of new isatin derivatives as drug targets^19^. Nintedanib (Ofev^®^) **II** (Figure 1), an orally available triple angiokinase inhibitor, received its first global approval in the US in October 2014 for the treatment of idiopathic pulmonary fibrosis (IPF)^20^. By 2015, Nintedanib **II** has been approved by the European medicines agency (EMA) in Europe, as a second-line treatment in combination with docetaxel of locally advanced, metastatic or locally recurrent non-small cell lung cancer of adenocarcinoma^20^.

On the other hand, quinazolines constitute a leading class of heterocycles that displayed interesting biological activities, chiefly anticancer activity. Raltitrexed (Tomudex^®^) **III**, Afatinib (Gilotrif^®^) **IV** and Gefitinib (Iressa^®^) **V** (Figure 1) are examples for the clinically approved quinazoline-based anticancer drugs^21^. Besides, phthalazine nucleus has emerged as a promising and attractive one in the development of novel anticancer agents^22–24^. Olaparib (Lynparza^®^) **VI** (Figure 1) is an oral small molecule phthalazine-based poly ADP-ribose polymerase (PARP) inhibitor being developed for the treatment of solid tumors. In December 2014, Olaparib was approved in the EU and USA for the treatment of BRCA-mutated ovarian cancer^25^.

**Figure 2.**
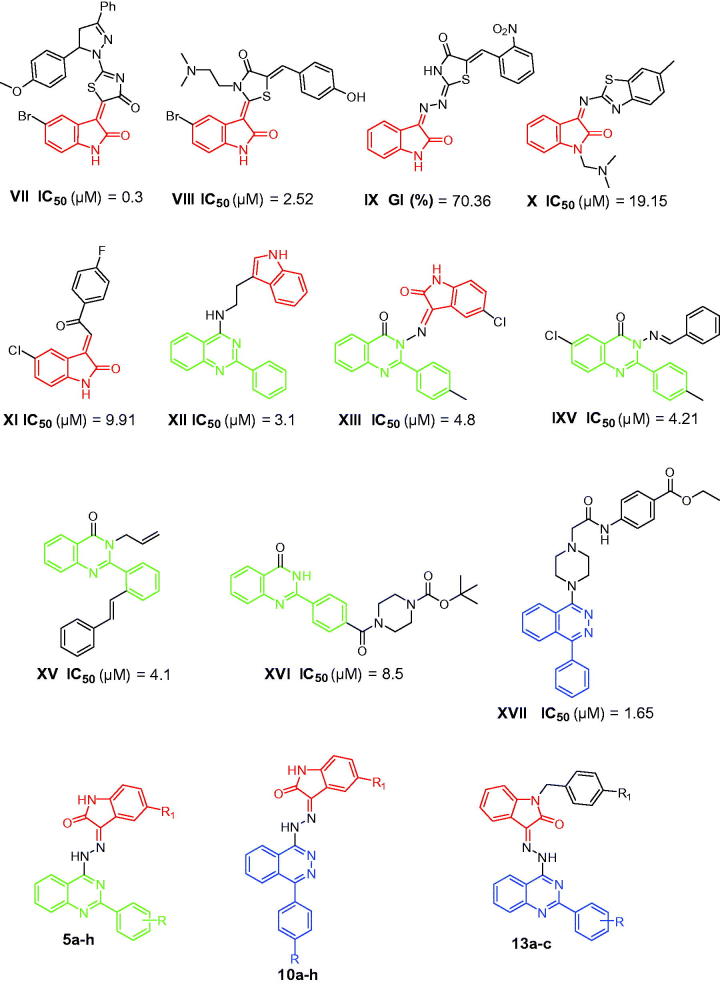
Structures of some reported isatins, quinazolines and phthalazines **VII**–**XVII** with anti-proliferative activity against the triple-negative breast cancer MDA-MB-231 cells, and structures of the target hybrids **5a–h**, **10a–h** and **13a–c**.

Surveying the literature revealed that different isatin^26–30^, 2-phenylquinazoline^31–35^ and 4-arylphthalazine^23^^,^^36–38^ derivatives were developed with significant activity against the TNBC MDA-MB-231 cell line. Figure 2 displays some of these derivatives with their IC_50_ values or growth inhibition (GI) percentage against MDA-MB-231 cells.

In light of the above findings and as a part of our ongoing effort to develop potent isatin-based anti-proliferative agents^39–45^, we utilized the hybrid pharmacophore approach to design and synthesize three different series of novel isatin-based hybrids **5a–h**, **10a–h** and **13a–c** (Figure 2), *via* merging the pharmacophoric elements of isatin and phthalazine or quinazoline in a single chemical framework through a hydrazine linker, with the prime goal of developing potent anti-proliferative agents against the TNBC MDA-MB-231 cell line. The three synthesized series were *in vitro* evaluated for their potential anti-proliferative activity against TNBC MDA-MB-231 cell line. Compounds **5e** and **10g** were further estimated for their apoptosis induction potential in MDA-MB-231 cells, to gain mechanistic insights into the anti-proliferative activity of the prepared hybrids. Furthermore, the activities against A549 alveolar carcinoma, Caco-2 colon cancer, LoVo human colorectal carcinoma and HepG2 hepatocellular carcinoma were *in vitro* examined.

## Materials and methods

### Chemistry

Melting points were measured with a Stuart melting point apparatus (Bibby Scientific Limited, Staffordshire, UK) and were uncorrected. Infrared spectra were recorded as potassium bromide discs on Schimadzu FT-IR 8400S spectrophotometer (Shimadzu Scientific Instruments, Kyoto, Japan) and expressed in wave number (cm^−1^). The NMR spectra were recorded on Varian Gemini-300BB 300 MHz FT-NMR spectrometers (Varian Inc., Palo Alto, CA). ^1^H and ^13^C spectra were run at 300 and 75 MHz, respectively, in deuterated dimethyl sulfoxide (DMSO-d_6_). Chemical shifts (*δ*_H_) are reported relative to tetramethylsilane (TMS) as internal standard. All coupling constant (*J*) values are given in hertz. Chemical shifts (*δ*_C_) are reported relative to DMSO-d_6_ as internal standards. The abbreviations used are as follows: s, singlet; d, doublet; m, multiplet. Elemental analyses were carried out at the Regional Center for Microbiology and Biotechnology, Al-Azhar University, Cairo, Egypt. Reaction courses and product mixtures were routinely monitored by thin layer chromatography (TLC) on silica gel precoated F_254_ Merck plates (Merck Group, Darmstadt, Germany). Unless otherwise noted, all solvents and reagents were commercially available and used without further purification.

#### *General procedure for preparation of 4-chloroquinazolines* 2a,b

To a cooled stirred mixture of phosphorus oxychloride (30 mL) and *N*,*N*-dimethylaniline (1 mL), 2-Substituted-quinazolin-4(3*H*)-ones **1a**,**b** (10 mmol) were added portion-wise. After refluxing for 6 h, the reaction mixture was cooled then poured onto ice-water and alkalinized with 2 N NaOH. The aqueous solution was extracted with methylene chloride. The combined organic layer was dried over anhydrous Na_2_SO_4_ then filtered, and the solvent was removed under reduced pressure. The obtained solid was crystallized from isopropanol to afford compounds **2a**,**b**^46^.

#### *General procedure for preparation of 4-hydrazinyl-2-substituted quinazoline* 3a,b

To a magnetically stirred solution of 4-chloroquinazolines **2a,b** (5 mmol) in ethanol (15 mL**),** hydrazine hydrate 99% (2.5 mL, 50 mmol) was added. The stirring was continued at refluxing temperature for 4 h. Upon cooling, the obtained precipitate was filtered, washed with methanol and water, dried and recrystallized from ethanol to furnish compounds **3a,b**.

2–(2,6-Dichlorophenyl)-4-hydrazinylquinazoline (**3b**). White crystals (yield 86%), m.p. 237–239 °C; IR (KBr, *ν* cm^−1^): 3295 (NH, NH_2_) cm^−1^; ^1^H NMR (DSMO-d_6_) *δ ppm:* 4.77 (s, 2H, NH_2_, D_2_O exchangeable), 7.51–7.54 (m, 3H, Ar–H), 7.69–7.87 (m, 3H, Ar–H), 8.21 (d, 1H, Ar–H, *J* = 8.1 Hz), 9.77 (s, 1H, NH, D_2_O exchangeable); Anal. Calcd. for C_14_H_10_Cl_2_N_4_:C, 55.10; H, 3.30; N, 18.36; Found C, 54.89; H, 3.33; N, 18.41.

#### *General procedure for preparation of the targetisatin-quinazoline hybrids* 5a–h

To a stirred solution of hydrazine derivatives **3a,b** (1 mmol), in absolute ethyl alcohol, the appropriate isatin **4a–d** (1 mmol) and catalytic amount of glacial acetic acid were added. The mixture was heated under reflux for 0.5 h, filtered while hot. The obtained solid was washed with ethanol collected and crystallized from an ethanol/DMF mixture to obtain hybrids **5a–h**.

*3–(2–(2–(4-Chlorophenyl)quinazolin-4-yl)hydrazono)indolin-2-one* (**5a**). Orange powder (yield 75%), m.p. 291–292 °C; IR (KBr, *ν* cm^−1^): 3210 (NH) and 1675 (C = O); ^1^H NMR (300 MHz, DMSO-d_6_) *δ ppm*: 6.98 (d, 1H, Ar–H, *J* = 7.8 Hz), 7.14 (t, 1H, Ar–H, *J* = 7.5 Hz), 7.38 (t, 1H, Ar–H, *J* = 7.8 Hz), 7.61 (d, 2H, H-3 and H-5 of 4-Cl-C_6_H_4_, *J* = 8.4 Hz), 7.69–7.76 (m, 3H, Ar–H), 7.98–8.00 (m, 2H, Ar–H), 8.54 (d, 2H, H-2 and H-6 of 4-Cl-C_6_H_4_, *J* = 8.7 Hz), 11.38 (s, 1H, NH, D_2_O exchangeable), 13.89 (s, 1H, NH, D_2_O exchangeable); ^13^C NMR (75 MHz, DMSO-d_6_) *δ*: 111.23, 112.70, 120.00, 120.62, 122.63, 123.37, 127.46, 128.49, 128.66, 129.72, 131.19, 134.11, 135.48, 136.24, 141.83, 151.51, 156.15, 157.99, 163.29 (C = O); Anal. Calcd. for C_22_H_14_ClN_5_O: C, 66.09; H, 3.53; N, 17.52; Found C, 66.21; H, 3.49; N, 17.45.

*3–(2–(2–(4-Chlorophenyl)quinazolin-4-yl)hydrazono)-5-fluoroindolin-2-one* (**5b**). Orange powder (yield 77%), m.p. >300 °C; IR (KBr, *ν* cm^−1^): 3190 (NH) and 1690 (C = O); ^1^H NMR (300 MHz, DMSO-d_6_) *δ ppm*: 6.95 (dd, 1H, Ar–H, *J* = 4.2, 8.7 Hz), 7.19 (t, 1H, Ar–H, *J* = 8.7 Hz), 7.59–7.63 (m, 3H, Ar–H), 7.71–7.77 (m, 2H, Ar–H), 7.96 (d, 2H, Ar–H, *J* = 7.8 Hz), 8.53 (d, 2H, H-2 and H-6 of 4-Cl-C_6_H_4_, *J* = 8.4 Hz), 11.39 (s, 1H, NH, D_2_O exchangeable), 13.86 (s, 1H, NH, D_2_O exchangeable); ^13^C NMR (75 MHz, DMSO-d_6_) *δ*: 107.93, 112.72, 116.75, 121.22, 127.43, 128.54, 129.78, 133.67, 134.29, 135.54, 136.16, 137.95, 138.07, 151.61, 156.13, 157.95, 163.44, 167.34 (C = O); Anal. Calcd. for C_22_H_13_ClFN_5_O: C, 63.24; H, 3.14; N, 16.76; Found C, 63.39; H, 3.11; N, 16.68.

*5-Chloro-3–(2–(2–(4-chlorophenyl)quinazolin-4-yl)hydrazono)indolin-2-one* (**5c**). Orange powder (yield 70%), m.p. > 300 °C; IR (KBr, *ν* cm^−1^): 3285 (NH) and 1672 (C = O); ^1^H NMR (300 MHz, DMSO-d_6_) *δ ppm*: 6.98 (d, 1H, Ar–H, *J* = 8.4 Hz), 7.41 (d, 1H, Ar–H, *J* = 8.4 Hz), 7.61 (d, 2H, H-3 and H-5 of 4-Cl-C_6_H_4_, *J* = 8.4 Hz), 7.69–7.77 (m 3H, Ar–H), 7.97 (d, 2H, Ar, *J* = 7.8 Hz), 8.53 (d, 2H, H-2 and H-6 of 4-Cl-C_6_H_4_, *J* = 8.7 Hz), 11.49 (s, 1H, NH, D_2_O exchangeable), 13.86 (s, 1H, NH, D_2_O exchangeable); ^13^C NMR (75 MHz, DMSO-d_6_) *δ*: 112.68, 119.95, 120.10, 121.67, 123.47, 126.75, 127.50, 128.49, 128.68, 129.72, 134.29, 135.51, 136.11, 140.41, 151.58, 156.02, 157.90, 163.08 (C = O); Anal. Calcd. for C_22_H_13_Cl_2_N_5_O: C, 60.85; H, 3.02; N, 16.13**;** Found C, 60.97; H, 2.98; N, 16.05.

*5-Bromo-3–(2–(2–(4-chlorophenyl)quinazolin-4-yl)hydrazono)indolin-2-one* (**5d**). Orange powder (yield 77%), m.p. >300 °C; IR (KBr, *ν * cm^−1^): 3245 (NH) and 1679 (C = O); ^1^H NMR (300 MHz, DMSO-d_6_) *δ ppm*: 6.94 (d, 1H, Ar–H, *J* = 8.1 Hz), 7.54 (dd, 1H, Ar–H, *J* = 2.1, 8.1 Hz), 7.61 (d, 2H, H-3 and H-5 of 4-Cl-C_6_H_4_, *J* = 8.7 Hz), 7.69–7.79 (m 2H, Ar–H), 7.87 (s, 1H, Ar–H), 7.98 (d, 2H, Ar–H, *J* = 3.9 Hz), 8.56 (d, 2H, H-2 and H-6 of 4-Cl-C_6_H_4_, *J* = 8.7 Hz), 11.49 (s, 1H, NH, D_2_O exchangeable), 13.88 (s, 1H, NH, D_2_O exchangeable); Anal. Calcd. for C_22_H_13_BrClN_5_O: C, 55.20; H, 2.74; N, 14.63; Found C, 55.33; H, 2.71; N, 14.54.

*3–(2–(2–(2,6-Dichlorophenyl)quinazolin-4-yl)hydrazono)indolin-2-one* (**5e**). Orange powder (yield 65%), m.p. > 300 °C; IR (KBr, *ν* cm^−1^): 3170 (NH) and 1683 (C = O); ^1^H NMR (300 MHz, DMSO-d_6_) *δ ppm*: 6.88 (d, 1H, Ar–H, *J* = 7.5 Hz), 7.08 (t, 1H, Ar–H, *J* = 7.5 Hz), 7.31 (t, 1H, Ar–H, *J* = 7.5 Hz), 7.58–7.84 (m, 5H, Ar–H), 8.01 (s, 1H, Ar–H), 8.42 (d, 2H, Ar–H, *J* = 7.5 Hz), 10.62, 11.38 (s, 1H, NH, D_2_O exchangeable), 12.11, 13.93 (s, 1H, NH, D_2_O exchangeable); Anal. Calcd. for C_22_H_13_Cl_2_N_5_O: C, 60.85; H, 3.02; N, 16.13; Found 60.97; H, 2.99; N, 16.03.

*3–(2–(2–(2,6-Dichlorophenyl)quinazolin-4-yl)hydrazono)-5-fluoroindolin-2-one* (**5f**). Orange powder (yield 70%), m.p. > 300 °C; IR (KBr, *ν* cm^−1^): 3185 (NH) and 1675 (C = O); ^1^H NMR (300 MHz, DMSO-d_6_) *δ ppm*: 6.85 (dd, 1H, Ar–H, *J* = 4.5, 8.7 Hz), 7.20 (t, 1H, Ar–H, *J* = 8.7 Hz), 7.55–7.87 (m, 6H, Ar–H), 8.03 (s, 1H, Ar–H), 8.45 (d, 1H, Ar–H, *J* = 8.4 Hz), 10.65, 11.39 (s, 1H, NH, D_2_O exchangeable), 12.25, 13.91 (s, 1H, NH, D_2_O exchangeable); ^13^C NMR (75 MHz, DMSO-d_6_) *δ*: 112.37, 117.99, 121.07, 123.75, 127.34, 128.35, 129.42, 132.76, 134.26, 135.28, 136.92, 138.10, 139.76, 146.37, 151.24, 155.92, 156.80, 158.99, 159.42, 159.95, 163.40, 165.44 (C = O); Anal. Calcd. for C_22_H_12_Cl_2_FN_5_O: C, 58.43; H, 2.67; N, 15.49; Found C, 58.29; H, 2.71; N, 15.58.

*5-Chloro-3–(2–(2–(2,6-dichlorophenyl)quinazolin-4-yl)hydrazono)indolin-2-one* (**5g**). Orange powder (yield 75%), m.p. >300 °C; IR (KBr, *ν* cm^−1^): 3203 (NH) and 1688 (C = O); ^1^H NMR (300 MHz, DMSO-d_6_) *δ ppm*: 6.89 (d, 1H, Ar–H, *J* = 8.4 Hz), 7.37 (t, 1H, Ar–H, *J* = 8.7 Hz), 7.58 (d, 1H, Ar–H, *J* = 8.4 Hz), 7.72–7.88 (m, 5H, Ar–H), 8.01 (s, 1H, Ar–H), 8.39 (d, 1H, Ar–H, *J* = 8.4 Hz), 10.75, 11.48 (s, 1H, NH, D_2_O exchangeable), 12.37, 13.85 (s, 1H, NH, D_2_O exchangeable); Anal. Calcd. for C_22_H_12_Cl_3_N_5_O: C, 56.37; H, 2.58; N, 14.94; Found C, 56.51; H, 2.53; N, 14.82.

*5-Bromo-3–(2–(2–(2,6-dichlorophenyl)quinazolin-4-yl)hydrazono)indolin-2-one* (**5h**). Orange powder (yield 72%), m.p. >300 °C; IR (KBr, *ν* cm^−1^): 3305 (NH) and 1670 (C = O); ^1^H NMR (300 MHz, DMSO-d_6_) *δ ppm*: 6.84 (d, 1H, Ar–H, *J* = 8.7 Hz), 7.49–8.02 (m, 7H, Ar–H), 8.36 (d, 1H, Ar–H, *J* = 8.4 Hz), 8.53 (s, 1H, Ar–H), 10.76, 11.48 (s, 1H, NH, D_2_O exchangeable), 12.15, 13.85 (s, 1H, NH, D_2_O exchangeable); Anal. Calcd. for C_22_H_12_BrCl_2_N_5_O: C, 51.49; H, 2.36; N, 13.65; Found C, 51.30; H, 2.39; N, 13.71.

#### *1-Chloro-4-phenylphthalazines* 8a,b

Compounds **8a,b** were prepared according to the literature procedure^47^^,^^48^.

#### General procedure for preparation of 1-hydrazinyl-4-phenylphthalazines 9a,b

Compounds **9a**,**b** were prepared according to the literature procedure^49^.

#### *General procedure for preparation of the target isatin-phthalazine hybrids* (10a–h)

Hydrazino phthalazines **9a,b** (1 mmol) and catalytic amount of glacial acetic acid were added to a stirred solution of indoline-2,3-dione **4a–d** (1 mmol) in refluxing absolute ethyl alcohol, then the reaction mixture was refluxed for 1 h. The precipitated solid was filtered while hot, dried and recrystallized from ethanol/DMF mixture to afford the target hybrids **10a–h**.

*3–(2–(4-Phenylphthalazin-1-yl)hydrazono)indolin-2-one* (**10a**). Orange powder (yield 65%), m.p. > 300 °C; IR (KBr, *ν* cm^−1^): 3183 (NH) and 1677 (C = O); ^1^H NMR (300 MHz, DMSO-d_6_) *δ ppm*: 6.87 (d, 1H, H-7 isatin, *J* = 7.8 Hz), 7.05 (t, 1H, H-5 isatin, *J* = 7.5 Hz), 7.29 (t, 1H, H-6 isatin, *J* = 7.5 Hz), 7.56–7.63 (m, 5H, H-2, H-3, H-4, H-5 & H-6 of C_6_H_5_), 7.68 (d, 1H, H-8 phthalazine, *J* = 8.1 Hz), 7.89 (t, 1H, H-6 phthalazine, *J* = 7.8 Hz), 7.96 (t, 1H, H-7 phthalazine, *J* = 7.8 Hz), 8.48 (d, 1H, H-4 isatin, *J* = 7.8 Hz), 8.68 (d, 1H, H-5 phthalazine, *J* = 7.8 Hz), 10.56 (s, 1H, NH, D_2_O exchangeable), 12.83 (s, 1H, NH, D_2_O exchangeable); Anal. Calcd. for C_22_H_15_N_5_O: C, 72.32; H, 4.14; N, 19.17; Found C, 72.44; H, 4.11; N, 19.12.

*5-Fluoro-3–(2–(4-phenylphthalazin-1-yl)hydrazono)indolin-2-one* (**10b**). Orange powder (yield 60%), m.p. >300 °C; IR (KBr, *ν* cm^−1^): 3234 (NH) and 1677 (C = O); ^1^H NMR (300 MHz, DMSO-d_6_) *δ ppm*: 6.86 (dd, 1H, H-6 isatin, *J* = 4.2, 8.7 Hz), 7.14 (t, 1H, H-7 isatin, *J* = 8.4 Hz), 7.57–7.64 (m, 5H, H-2, H-3, H-4, H-5 & H-6 of C_6_H_5_), 7.71 (d, 1H, H-8 phthalazine, *J* = 7.8 Hz), 7.91 (t, 1H, H-6 phthalazine, *J* = 7.8 Hz), 7.96 (t, 1H, H-7 phthalazine, *J* = 7.8 Hz), 8.20 (dd, 1H, H-4 isatin, *J* = 3.0, 8.4 Hz), 8.68 (d, 1H, H-5 phthalazine, *J* = 7.5 Hz), 10.58 (s, 1H, NH, D_2_O exchangeable), 13.04 (s, 1H, NH, D_2_O exchangeable); ^13^C NMR (75 MHz, DMSO-d_6_) *δ*: 109.52, 113.88, 118.19, 123.64, 126.08, 126.62, 127.00, 128.54, 129.37, 132.69, 133.78, 134.55, 139.06, 143.32, 149.31, 151.86, 155.86, 158.98, 165.87, 171.92; Anal. Calcd. for C_22_H_14_FN_5_O: C, 68.92; H, 3.68; N, 18.27; Found C, 68.77; H, 3.73; N, 18.33.

*5-Chloro-3–(2–(4-phenylphthalazin-1-yl)hydrazono)indolin-2-one* (**10c**). Orange powder (yield 72%), m.p. >300 °C; IR (KBr, *ν* cm^−1^): 3195 (NH) and 1679 (C = O); ^1^H NMR (300 MHz, DMSO-d_6_) *δ ppm*: 6.89 (d, 1H, H-6 isatin, *J* = 8.4 Hz), 7.34 (d, 1H, H-7 isatin, *J* = 8.4 Hz), 7.57–7.64 (m, 5H, H-2, H-3, H-4, H-5 & H-6 of C_6_H_5_), 7.72 (d, 1H, H-8 phthalazine, *J* = 8.1 Hz), 7.92 (t, 1H, H-6 phthalazine, *J* = 8.1 Hz), 8.02 (t, 1H, H-7 phthalazine, *J* = 8.1 Hz), 8.45 (s, 1H, H-4 isatin), 8.62 (d, 1H, H-5 phthalazine, *J* = 8.1 Hz), 10.68 (s, 1H, NH, D_2_O exchangeable), 13.03 (s, 1H, NH, D_2_O exchangeable); Anal. Calcd. for C_22_H_14_ClN_5_O: C, 66.09; H, 3.53; N, 17.52; Found C, 66.21; H, 3.49; N, 17.42.

*5-Bromo-3–(2–(4-phenylphthalazin-1-yl)hydrazono)indolin-2-one* (**10d**). Orange powder (yield 75%), m.p. >300 °C; IR (KBr, *ν* cm^−1^): 3315 (NH) and 1672 (C = O); ^1^H NMR (300 MHz, DMSO-d_6_) *δ ppm*: 6.85 (d, 1H, H-6 isatin, *J* = 8.4 Hz), 7.47 (d, 1H, H-7 isatin, *J* = 8.1 Hz), 7.57–7.64 (m, 5H, H-2, H-3, H-4, H-5 & H-6 of C_6_H_5_), 7.72 (d, 1H, H-8 phthalazine, *J* = 8.4 Hz), 7.92 (t, 1H, H-6 phthalazine, *J* = 8.1 Hz), 8.01 (t, 1H, H-7 phthalazine, *J* = 8.1 Hz), 8.59–8.62 (m, 2H, H-4 isatin and H-5 phthalazine), 10.70 (s, 1H, NH, D_2_O exchangeable), 13.06 (s, 1H, NH, D_2_O exchangeable); Anal. Calcd. for C_22_H_14_BrN_5_O: C, 59.47; H, 3.18; N, 15.76; Found C, 59.61; H, 3.14; N, 15.68.

*3–(2–(4–(4-Chlorophenyl)phthalazin-1-yl)hydrazono)indolin-2-one* (**10e**). Yellow powder (yield 70%), m.p. >300 °C; IR (KBr, *ν* cm^−1^): 3218 (NH) and 1676 (C = O); ^1^H NMR (300 MHz, DMSO-d_6_) *δ ppm*: 6.87 (d, 1H, H-7 isatin, *J* = 7.8 Hz), 7.05 (t, 1H, H-5 isatin, *J* = 7.5 Hz), 7.29 (t, 1H, H-6 isatin, *J* = 7.5 Hz), 7.60–7.65 (m, 4H, H-2, H-3, H-5 & H-6 of 4-Cl-C_6_H_5_), 7.67 (d, 1H, H-8 phthalazine, *J* = 7.8 Hz), 7.89 (t, 1H, H-6 phthalazine, *J* = 7.8 Hz), 7.96 (t, 1H, H-7 phthalazine, *J* = 7.8 Hz), 8.47 (d, 1H, H-4 isatin, *J* = 7.2 Hz), 8.68 (d, 1H, H-5 phthalazine, *J* = 7.5 Hz), 10.63 (s, 1H, NH, D_2_O exchangeable), 13.04 (s, 1H, NH, D_2_O exchangeable); Anal. Calcd. for C_22_H_14_ClN_5_O: C, 66.09; H, 3.53; N, 17.52; Found C, 65.89; H, 3.58; N, 17.61.

*3–(2–(4–(4-Chlorophenyl)phthalazin-1-yl)hydrazono)-5-fluoroindolin-2-one* (**10f**). Orange powder (yield 73%), m.p. >300 °C; IR (KBr, *ν* cm^−1^): 3247 (NH) and 1670 (C = O); ^1^H NMR (300 MHz, DMSO-d_6_) *δ ppm*: 6.86 (dd, 1H, H-6 isatin, *J* = 4.2, 8.1 Hz), 7.13 (t, 1H, H-7 isatin, *J* = 8.1 Hz), 7.64–7.66 (m, 4H, H-2, H-3, H-5 & H-6 of 4-Cl-C_6_H_5_), 7.70 (d, 1H, H-8 phthalazine, *J* = 8.1 Hz), 7.92 (t, 1H, H-6 phthalazine, *J* = 7.8 Hz), 8.01 (t, 1H, H-7 phthalazine, *J* = 7.8 Hz), 8.20 (dd, 1H, H-4 isatin, *J* = 3.0, 8.4 Hz), 8.66 (d, 1H, H-5 phthalazine, *J* = 7.8 Hz), 10.58 (s, 1H, NH, D_2_O exchangeable), 13.05 (s, 1H, NH, D_2_O exchangeable); Anal. Calcd. for C_22_H_13_ClFN_5_O: C, 63.24; H, 3.14; N, 16.76; Found C, 63.08; H, 3.09; N, 16.82.

*5-Chloro-3–(2–(4–(4-chlorophenyl)phthalazin-1-yl)hydrazono)indolin-2-one* (**10g**). Orange powder (yield 70%), m.p. >300 °C; IR (KBr, *ν* cm^−1^): 3195 (NH) and 1673 (C = O); ^1^H NMR (300 MHz, DMSO-d_6_) *δ ppm*: 6.90 (d, 1H, H-6 isatin, *J* = 8.1 Hz), 7.35 (d, 1H, H-7 isatin, *J* = 8.4 Hz), 7.66–7.69 (m, 4H, H-2, H-3, H-5 & H-6 of 4-Cl-C_6_H_5_), 7.70 (d, 1H, H-8 phthalazine, *J* = 8.1 Hz), 7.92 (t, 1H, H-6 phthalazine, *J* = 8.1 Hz), 8.01 (t, 1H, H-7 phthalazine, *J* = 7.8 Hz), 8.43 (s, 1H, H-4 isatin), 8.61 (d, 1H, H-5 phthalazine, *J* = 7.8 Hz), 10.69 (s, 1H, NH, D_2_O exchangeable), 13.12 (s, 1H, NH, D_2_O exchangeable); ^13^C NMR (75 MHz, DMSO-d_6_) *δ*: 111.44, 118.91, 125.29, 126.05, 126.35, 126.87, 128.75, 130.13, 131.32, 133.40, 133.84, 134.07, 141.45, 142.72, 148.30, 151.91, 165.62; Anal. Calcd. for C_22_H_13_Cl_2_N_5_O: C, 60.85; H, 3.02; N, 16.13; Found C, 61.03; H, 2.97; N, 16.06.

*5-Bromo-3–(2–(4–(4-chlorophenyl)phthalazin-1-yl)hydrazono)indolin-2-one* (**10h**). Yellow powder (yield 75%), m.p. >300 °C; IR (KBr, *ν* cm^−1^): 3210 (NH) and 1678 (C = O); ^1^H NMR (300 MHz, DMSO-d_6_) *δ ppm*: 6.85 (d, 1H, H-6 isatin, *J* = 8.1 Hz), 7.47 (d, 1H, H-7 isatin, *J* = 8.4 Hz), 7.66–7.69 (m, 4H, H-2, H-3, H-5 & H-6 of 4-Cl-C_6_H_5_), 7.71 (d, 1H, H-8 phthalazine, *J* = 7.8 Hz), 7.92 (t, 1H, H-6 phthalazine, *J* = 8.1 Hz), 8.01 (t, 1H, H-7 phthalazine, *J* = 7.8 Hz), 8.58–8.61 (m, 2H, H-4 isatin and H-5 phthalazine), 10.74 (s, 1H, NH, D_2_O exchangeable), 13.17 (s, 1H, NH, D_2_O exchangeable); ^13^C NMR (75 MHz, DMSO-d_6_) *δ*: 111.83, 113.01, 119.40, 123.42, 125.07, 126.06, 126.89, 128.66, 129.11, 131.32, 132.69, 133.39, 133.85, 134.07, 141.80, 142.60, 148.30, 151.89, 165.49; Anal. Calcd. for C_22_H_13_BrClN_5_O: C, 55.20; H, 2.74; N, 14.63; Found C, 55.36; H, 2.77; N, 14.55.

#### N-*Benzylindoline-2,3-diones****12a,b***

Compounds **12a**,**b** were prepared according to the literature procedure^45^.

##### *General procedure for preparation of the target hybrids* (13a-c)

Following the same procedures described for preparation of the hybrids **5a–h**, using *N*-benzylindoline-2,3-diones **7a,b** instead of indoline-2,3-dione **4a–d**.

*1-Benzyl-3–(2–(2–(4-chlorophenyl)quinazolin-4-yl)hydrazono)indolin-2-one* (**13a**). Orange powder (yield 65%), m.p. 291–293 °C; IR (KBr, *ν* cm^−1^): 1681 (C = O); ^1^H NMR (300 MHz, DMSO-d_6_) *δ ppm*: 5.00, 5.06 (s, 2H, –CH_2_–), 6.99 (d, 1H, Ar–H, *J* = 7.8 Hz), 7.16 (t, 1H, Ar–H, *J* = 7.5 Hz), 7.27–7.46 (m, 6H, Ar–H), 7.59–7.98 (m, 6H, Ar), 8.10 (d, 1H, Ar–H, *J* = 7.5 Hz), 8.47–8.57 (m, 2H, Ar–H), 11.42, 13.78 (s, 1H, NH, D_2_O exchangeable); Anal. Calcd. for C_29_H_20_ClN_5_O: C, 71.09; H, 4.11; N, 14.29; Found C, 70.91; H, 4.16; N, 14.21.

*1-Benzyl-3–(2–(2–(2,6-dichlorophenyl)quinazolin-4-yl)hydrazono)indolin-2-one* (**13b**). Orange powder (yield 70%), m.p. >300 °C; IR (KBr, *ν* cm^−1^): 1674 (C = O); ^1^H NMR (300 MHz, DMSO-d_6_) *δ ppm*: 4.98, 5.07 (s, 2H, –CH_2_–), 6.98 (d, 1H, Ar–H, *J* = 7.5 Hz), 7.10 (t, 1H, Ar–H, *J* = 7.8 Hz), 7.25–7.37 (m, 5H, Ar–H), 7.42 (t, 1H, Ar–H, *J* = 7.8 Hz), 7.61 (d, 1H, Ar–H, *J* = 7.8 Hz), 7.68–7.89 (m, 4H, Ar–H), 8.03 (d, 1H, Ar–H, *J* = 8.4 Hz), 8.51–8.53 (m, 2H, Ar–H), 12.28, 13.87 (s, 1H, NH, D_2_O exchangeable); Anal. Calcd. for C_29_H_19_Cl_2_N_5_O: C, 66.42; H, 3.65; N, 13.36; Found C, 66.57; H, 3.69; N, 13.28.

*3–(2–(2–(4-Chlorophenyl)quinazolin-4-yl)hydrazono)-1–(4-fluorobenzyl) indolin-2-one* (**13c**). Orange powder (yield 68%), m.p. > 300 °C; IR (KBr, *ν* cm^−1^): 1677 (C = O); ^1^H NMR (300 MHz, DMSO-d_6_) *δ ppm*: 4.99, 5.05 (s, 2H–CH_2_–), 7.02 (d, 1H, Ar–H, *J* = 7.2 Hz), 7.12–7.19 (m, 4H, Ar–H), 7.30 (t, 1H, Ar–H, *J* = 7.5 Hz), 7.42–7.81 (m, 6H, Ar–H), 7.98 (s, 1H, Ar–H), 8.18 (d, 1H, Ar–H, *J* = 8.1 Hz), 8.48–8.56 (m, 2H, Ar–H), 11.67, 13.79 (s, 1H, NH, D_2_O exchangeable); Anal. Calcd. for C_29_H_19_ClFN_5_O: C, 68.57; H, 3.77; N, 13.79; Found C, 68.72; H, 3.81; N, 13.73.

### Biological evaluation

#### *In vitro* anti-proliferative activity

Human breast cancer (MDA-MB-231) cells, LoVo human colorectal carcinoma cells, human hepatoma (HepG2) cells and human non-small cell lung cancer (A549) cells were obtained from American Type Culture Collection (ATCC). Cells were maintained in Dulbecco’s Modified Eagle’s Medium (DMEM) (Sigma-Aldrich, St. Louis, MO), supplemented with 10% fetal bovine serum (Lonza Group, Basel, Switzerland), 100 IU/mL penicillin, 100 mg/mL streptomycin and 2 mmol/L L-glutamine (Sigma). Cells were seeded into 96-well plates at 0.4 × 10^4^/well and incubated overnight. The medium was replaced with fresh one containing the desired concentrations of the test compounds.

Anti-proliferative activity of the prepared hybrids against MDA-MB-231 cells was evaluated by the cell GI assay using 5-Fluorouracil (5-FU) as a standard treatment. This assay was conducted by the use of WST-1 reagent for determination of IC_50_ for each compound and the results are given in Table 1. After 48 h of incubation with the synthesized agents in the treatment groups or the vehicle (1% DMSO) in the control group, 10 μL of the WST-1 reagent were added to each well and the plates were re-incubated for 4 h at 37 °C. The amount of formazan was quantified using ELISA reader at 450 nm. Moreover, cytotoxicity of the compounds and the reference drug, doxorubicin in LoVo, HepG2 and A549 cells was assessed against control group at the end of exposure using the sulforhodamine B (SRB) assay. Experimental conditions were tested using three replicates (three wells of the 96-well plate per experimental condition) and all experiments were performed in triplicates. IC_50_ was calculated according to the equation for Boltzman sigmoidal concentration–response curve using the nonlinear regression fitting models by Graph Pad, Prism version 5 (GraphPad Software Inc., La Jolla, CA).

**Table 1.** *In vitro* anti-proliferative activity of the newly synthesized hybrids against MDA-MB-231 cell line.


Compound	R	R_1_	IC_50_ (μM)^a^MDA-MB-231
**5a**	4-Cl	H	13.18 ± 0.36
**5b**	4-Cl	F	15.15 ± 0.15
**5c**	4-Cl	Cl	17.93 ± 0.11
**5d**	4-Cl	Br	19.68 ± 0.26
**5e**	2,6-Cl_2_	H	12.35 ± 0.12
**5f**	2,6-Cl_2_	F	12.95 ± 0.31
**5g**	2,6-Cl_2_	Cl	14.59 ± 0.23
**5h**	2,6-Cl_2_	Br	17.31 ± 0.55
**10a**	H	H	13.96 ± 0.10
**10b**	H	F	15.62 ± 0.14
**10c**	H	Cl	17.63 ± 0.37
**10d**	H	Br	21.39 ± 0.13
**10e**	Cl	H	12.86 ± 0.12
**10f**	Cl	F	12.94 ± 0.23
**10g**	Cl	Cl	12.00 ± 0.13
**10h**	Cl	Br	15.21 ± 0.15
**13a**	4-Cl	H	26.21 ± 2.07
**13b**	2,6-Cl_2_	H	48.72 ± 2.59
**13c**	4-Cl	F	34.17 ± 2.42
**5-FU**			29.38 ± 1.24

a IC_50_ values are the mean ± SD of three separate experiments.

#### ELISA immunoassay

Effects of treatment of MDA-MB-231 cells with the most promising compounds **5e** and **10g** on the cells’ apoptotic machinery were further investigated. The levels of the apoptotic markers caspase-9, caspase-3 and Bax as well as the anti-apoptotic marker Bcl-2 were assessed using ELISA colorimetric kits (Neogen, Lexington, KY) as per the manufacturer’s instructions.

MDA-MB-231 cells were cultured as a monolayer in T-25 flasks and were seeded to attain 30% confluency prior to treatment. Cells were then treated separately with compounds **5e** and **10g** at their IC_50_ concentrations (12.35 and 12 μM, respectively) for 48 h. At the end of treatment, cells were collected *via* trypsinization and centrifuged at 10,000 rpm. The pellet was then rinsed with phosphate buffered saline (PBS) and lysed in radio immunoprecipitation assay (RIPA) lysis buffer at 4 °C for 45 min, then centrifuged at 14,000 rpm for 20 min to remove the cellular debris. Lysates were then collected and stored at −80 °C for later protein determination using Pierce BCA Protein Assay Kit (Pierce Biotechnology, Rockford, IL) according to manufacturer’s recommendations.

The cell lysate was diluted 10 times, and 100 μL (50 mg protein) was added to the wells of four separate microtiter plates for the four ELISA kits that were pre-coated with primary antibodies specific to caspase-9, caspase-3, Bax and Bcl-2 proteins, respectively. A secondary biotin-linked antibody specific to the protein captured by the primary antibody was further added to bind the captured protein, forming a “sandwich” of specific antibodies around the desired protein in the cell lysate. The streptavidin-horseradish peroxidase (HRP) complex was then used to bind the biotin-linked secondary antibody through its streptavidin portion. The HRP domain reacted with the added TMB substrate, forming a colored product that was measured at 450 nm by a plate reader (ChroMate-4300, Awareness Technology, Inc., Palm City, FL) after the reaction was terminated by the addition of stop solution.

#### Annexin V-FITC apoptosis

Apoptotic cells were further analyzed by Annexin V-FITC/DAPI assay (Cayman Chemical, Ann Arbor, MI). Briefly, MDA-MB-231 cells were cultured to a monolayer then treated with compound **10g** at the IC_50_ concentration (12 μM) as described earlier. Cells were then harvested *via* trypsinization, and rinsed twice in PBS followed by binding buffer. Moreover, cells were re-suspended in 100 μL of binding buffer with the addition of 1 μL of FITC-Annexin V (Becton Dickinson BD Pharmingen™, Heidelberg, Germany) followed by an incubation period of 30 min at 4 °C. Cells were then rinsed in binding buffer and re-suspended in 150 μL of binding buffer with the addition of 1 μL of DAPI (1 μg/μL in PBS) (Invitrogen, Life Technologies, Darmstadt, Germany). Cells were then analyzed using the flow cytometer BD FACS Canto II (BD Biosciences San Jose, CA) and the results were interpreted with FlowJo7.6.4 software (Tree Star, FlowJo LLC, Ashland, OR).

#### Statistical analysis

Data are presented as means ± SD. Individual groups were compared using the two-tailed independent student’s t-test. Multiple group comparisons were carried out using one-way analysis of variance (ANOVA) followed by the Tukey–Kramer test for post-hoc analysis. Statistical significance was accepted at a level of *p* < 0.05. All statistical analyses were performed using GraphPad InStat software, version 3.05 (GraphPad Software, Inc., La Jolla, CA). Graphs were sketched using GraphPad Prism software, version 5.00 (GraphPad Software, Inc., La Jolla, CA).

#### ADME profiling

Absorption, distribution, metabolism and excretion (ADME) profiling for the prepared hybrids was carried out using Discovery Studio 2.5 (Accelrys, San Diego, CA). All the tested hybrids were drawn as a small library then prepared using ligand protocol to find the suitable orientation in 3D. ADME profiling was predicted for the designed library using ADME descriptors protocol.

## Results and discussion

### Chemistry

**Scheme 1.**
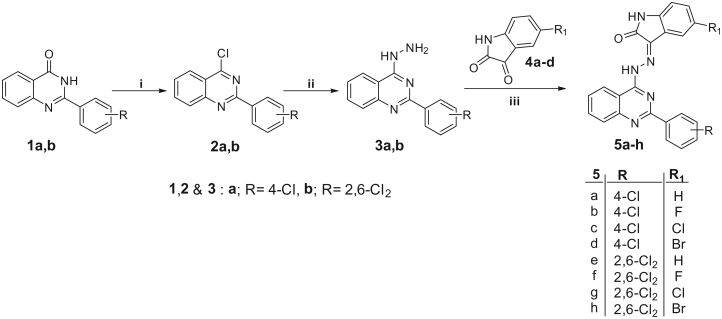
Reagents and conditions: **i**, POCl_3_/*N*,*N*-dimethylaniline/reflux 6 h; **ii**, NH_2_NH_2_.H_2_O/EtOH/reflux 4 h; **iii**, EtOH/AcOH (catalytic)/reflux 0.5 h.

**Scheme 2.**
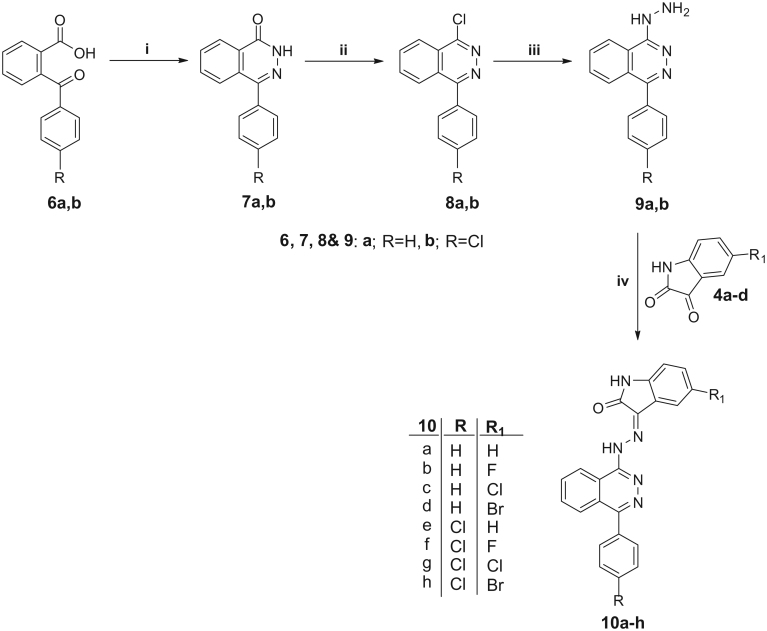
Reagents and conditions: **i**, NH_2_NH_2_.H_2_SO_4_/NaOH/reflux 1 h; **ii**, POCl_3_/*N*,*N*-dimethylaniline/reflux 6 h; **iii**, NH_2_NH_2_.H_2_O/EtOH/reflux 7 h. **iv**, EtOH/AcOH (catalytic)/reflux 1 h.

**Scheme 3.**
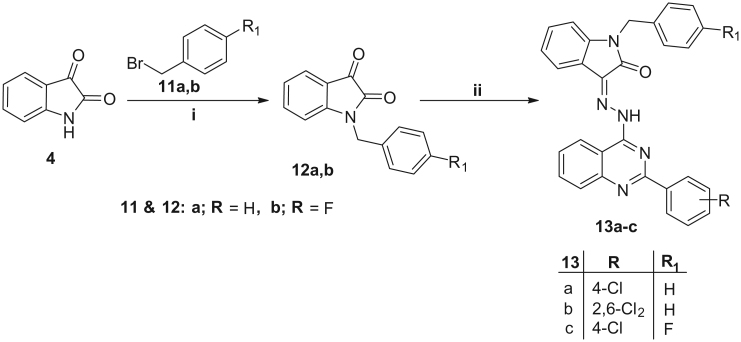
Reagents and conditions: **i**, DMF/K_2_CO_3_/reflux 3 h; **ii**, Compounds **4a,b**/EtOH/AcOH (catalytic)/reflux 0.5 h.

The synthetic strategies employed to prepare the new target hybrids are depicted in Schemes 1–3. In Scheme 1, 4-chloro-2-phenylquinazolines **2a,b** were obtained (in 75 and 72% yield, respectively), by chlorination of 2-phenylquinazolin-4(3*H*)-ones **1a,b** in excess refluxing phosphorous oxychloride. Next, 4-chloro-2-phenylquinazolines **2a,b** were refluxed with hydrazine hydrate in ethanol to furnish the hydrazine derivatives **3a,b** (in 81 and 86% yield, respectively), which were condensed with different isatins in refluxed ethanol in the presence of a catalytic amount of glacial acetic to afford the isatin-quinazoline hybrids **5a–h** (in 65–77% yield).

IR spectra of the latter hybrids displayed absorption bands due to the NH groups in the region 3170–3305 cm^−1^, in addition to a carbonyl band in the region 1672–1690 cm^−1^. The ^1^H NMR spectra of compounds **5a–h** confirmed the presence of two D_2_O-exchangeable singlet signals attributable to NH protons of the hydrazine function ( = N–NH–) and the isatin in the range *δ* 10.62–11.49 and 12.11–13.93 *ppm*, respectively.

For the synthesis of isatin-phthalazine hybrids **10a–h**, 2-benzoylbenzoic acids **6a,b** were cyclocondensed with hydrazine sulfate in the presence of sodium hydroxide to afford phthalazinone **7a,b**. Next, chlorination of compounds **7a,b** was carried out *via* refluxing with excess phosphorus oxychloride to furnish 1-chloro-4-phenylphthalazines **8a,b** (in 65 and 72% yield, respectively). The chloro intermediates **8a,b** were reacted with hydrazine hydrate in refluxing ethanol to provide the 1-hydrazinyl-4-phenylphthalazines **9a,b** (in 84 and 80% yield, respectively), which upon reaction with different isatins afforded the target hybrids **10a–h** (in 60–75% yield) (Scheme 2).

IR spectra of **10a–h** revealed the presence of an NH stretching band about 3200 cm^−1^. Their ^1^H NMR spectra showed two D_2_O-exchangeable singlet signals from the hydrazine function ( = N–NH–) and the isatin protons in the range *δ* 10.56–10.74 and 12.83–13.17 *ppm*.

Finally, isatin was refluxed with benzyl bromide or 4-fuorobenzyl bromide in dry DMF and anhydrous K_2_CO_3_ to furnish *N*-substituted isatins **12a,b,** respectively (in 88 and 82% yield, respectively). The reaction of derivatives **12a,b** with the appropriate 4-hydrazinyl-2-phenylquinazoline derivative 3 in ethanol in the presence of a catalytic amount of glacial acetic acid afforded the target hybrids **13a–c** (Scheme 3).

The structures of hybrids **13a–c** were confirmed under the basis of spectral and elemental analyses which were in full agreement with the proposed structures.

### Biological evaluation

#### *In vitro* anti-proliferative activity against TNBC MDA-MB-231 cell line

Anti-proliferative activity of the newly prepared hybrids **5a–h**, **10a-h** and **13a–c** was evaluated against MDA-MB-231 TNBC cell line using the WST-1 assay as described by Ngamwongsatit et al.^50^. Being a well-known broad-spectrum anticancer drug and a first line therapy for many tumors management, 5-FU was chosen as a positive control^51^,^52^. The anti-proliferative activity was expressed as the half maximal inhibitory concentration (IC_50_) values (Table 1).

From the obtained results, it is obvious that most of the tested hybrids have excellent to moderate growth inhibitory activity toward the tested MDA-MB-231 cancer cell line. In particular, compounds **5e** and **10g** were the most active members against MDA-MB-231 cells (IC_50_ = 12.35 ± 0.12 and 12.00 ± 0.13 μM, respectively), with 2.37- and 2.44-fold increased activity than the reference drug, 5-FU (IC_50_ = 29.38 ± 1.24 μM). Besides, compounds **5a–d**, **5f–h**, **10a–f**, **10h** and **13a** displayed good anti-proliferative activity with IC_50_ values ranging from 12.86 ± 0.12 to 26.21 ± 2.07 μM. Whilst, hybrids **13a** and **13b** were moderately active with IC_50_ values of 48.72 ± 2.59 and 34.17 ± 2.42 μM, respectively.

#### Structure activity relationship SAR

By scrutinizing the aforementioned biological data, one can snugly reveal a clear structure activity relationship. First, we investigated the impact of the substitution at the 5-position of the isatin moiety. Regarding the isatin-quinazoline hybrids **5a–h**, incorporation of unsubstituted isatin resulted in compounds **5a** and **5e** with moderate activity against the MDA-MB-231 cell line (IC_50_ = 13.18 ± 0.36 and 12.35 ± 0.12 μM, respectively). As it has an electronic and size properties similar to those of hydrogen, fluorine is introduced as a classical bioisostere to the hydrogen atom. Compounds **5b** and **5f** bearing fluorine substituent, displayed slight decrease in the anti-proliferative activity (IC_50_ = 15.15 ± 0.15 and 12.95 ± 0.31 μM, respectively) compared to the unsubstituted analogs, suggesting that halogens incorporation may not be advantageous for the activity. Furthermore, introduction of more bulky and lipophilic chlorine atom; compounds **5c** and **5g**, caused decrease of activity against MDA-MB-231 cells (IC_50_ = 17.9 ± 0.11 and 14.59 ± 0.23 μM, respectively). Also, incorporation of bromine atom (more bulky and lipophilic than chlorine) decreased the anti-proliferative activity (IC_50_ = 19.68 ± 0.26 and 17.31 ± 0.55 μM, respectively). Thus, the order of activity of the halogenated hybrids in such series, were smoothly decreased in the order of F > Cl > Br, hinting that increasing the lipophilicity and the size of the substituents of isatin moiety at the 5-position is not affirmative for the activity of the isatin-quinazoline hybrids regardless of the type of substitution of the 2-phenyl group of the quinazoline moiety.

Regarding the isatin-phthalazine hybrids **10a–h**, the above-mentioned relationship could be, also, proposed for the hybrids **10a–d** with unsubstituted 4-phenyl group in the phthalazine moiety. While, substitution of hybrids **10e–h** (containing 4–(4-chlorophenyl) group) with F or Cl at 5-position of isatin moiety has no significant effect, introduction of Br led to a decreased activity toward the MDA-MB-231 cell line.

Further examination of the effect of the substitution pattern of the phenyl group on the prepared hybrids was then conducted. Regarding the isatin-quinazoline hybrids **5a–h**, the increased IC_50_ values of members **5a–d** with incorporated 4-chlorophenyl group (13.18 ± 0.36, 15.15 ± 0.15, 17.93 ± 0.11 and 19.68 ± 0.26 μM, respectively) than that of their corresponding analogs **5e–h** with 2,6-dichlorophenyl group (12.35 ± 0.12, 12.95 ± 0.31, 14.59 ± 0.23 and 17.31 ± 0.55 μM, respectively) indicated that 2,6-dichloro substitution is more beneficial for activity rather than 4-chloro substitution, which could be attributed to the non-coplanarity or the increased lipophilicity. On the other hand, substitution of the 4-phenyl group in the isatin-phthalazine hybrids was found to be advantageous to the activity rather than unsubstitution, that is evidenced by the decreased IC_50_ values of compounds **10e–f** (12.86 ± 0.12, 12.94 ± 0.23, 12.00 ± 0.13 and 15.21 ± 0.15 μM, respectively) than those of their corresponding analogs **10a–d** (13.96 ± 0.10, 15.62 ± 0.14 17.63 ± 0.37 and 21.39 ± 0.13 μM, respectively).

Finally, investigation of the effect of N-benzylation of isatin moiety showed that this was not an advantageous approach for the anti-proliferative activity against MDA-MB-231 cell line, where the N-benzyl hybrids **13a–c** displayed decreased potency (26.21 ± 2.07, 48.72 ± 2.59 and 34.17 ± 2.42 μM, respectively) than their unsubstituted counterparts **5a** and **5e** (IC_50_ = 13.18 ± 0.36 and 12.35 ± 0.12 μM, respectively).

In conclusion, we can deduce that unsubstitution of isatin moiety at C-5 or N-1 positions, disubstitution of the 2-phenyl group of quinazoline moiety and substitution of the 4-phenyl group of phthalazine moiety are crucial elements for the anti-proliferative activity against MDA-MB-231.

#### Effects on mitochondrial apoptosis pathway proteins Bax andBcl-2

Induction of apoptosis in cancer cells is one of the successful strategies for the development of cancer therapy^53–55^. Therefore, we investigated the potential pro-apoptotic effects of our isatin-quinazoline and isatin-phthalazine hybrids attempting to explore the underlying mechanism for their anti-proliferative activity.

As indicated by the cytotoxicity results, compounds **5e** and **10g** were found to be the most active agents against MDA-MB-231 breast cancer cells. Thus, we investigated the potential pro-apoptotic effects of these promising agents in an attempt to define the principle mechanism for their anti-proliferative activity.

**Figure 3.**
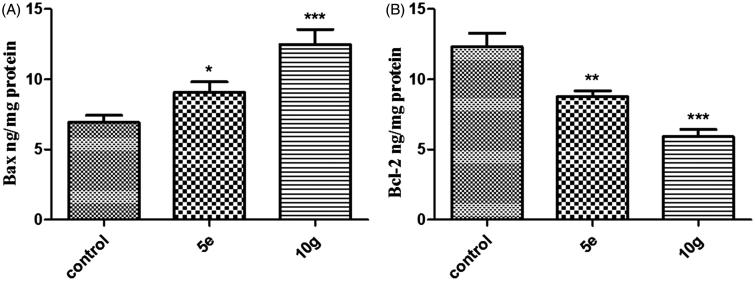
Effect of compounds 5e and 10g on the protein levels of A) Bax; B) Bcl-2 in MDA-MB-231 cells treated with the compounds at their IC_50_ concentrations against control (1% DMSO). Data are mean ± SD (*n* = 3). The experiment was done in triplicates. *Significantly different from control at *p* < 0.05. **Significantly different from control at *p* < 0.01. ***Significantly different from control at *p* < 0.001.

The Bcl-2 family of proteins is responsible for synchronizing the mitochondrial apoptotic pathway^56^. These proteins are classified into two groups: anti-apoptotic proteins such as Bcl-2 protein and the counteracting pro-apoptotic proteins including Bax protein^57^. It has been previously indicated that the overexpression of the anti-apoptotic Bcl-2 protein is a contributing factor to tumorigenesis in a multitude of cancers including breast cancer^58^. In this study, exposure of MDA-MB-231 breast cancer cells to compounds **5e** and **10g** for 48 h resulted in a significant increase in the expression of the pro-apoptotic protein Bax by ∼130 and 180%, respectively, compared to the control (Figure 3(A), Table 2). On the other hand, treatment of MDA-MB-231 breast cancer cells with compounds **5e** and **10g** significantly reduced the expression levels of the anti-apoptotic protein Bcl-2 by ∼29 and 52%, respectively, compared to the control (Figure 3(B), Table 2). A rather more important parameter is the ratio between Bax (apoptosis inducer) and Bcl-2 (apoptosis suppressor) that gave more real insight to the apoptotic activity of the compounds as it is considered a key indicator of therapeutic response to chemotherapy. Analyzing the results discloses that compound **5e** increased the Bax/Bcl-2 ratio two folds compared to the control, while compound **10g** even boosted it four folds in comparison to the control. The ability of the two compounds to down regulate Bcl-2 levels while boosting Bax levels further supports their effectiveness as apoptosis inducers.

**Table 2.** Effect of compounds **5e** and **10g** on active caspase-9 and -3 levels, and the expression levels of Bcl-2 and Bax in MDA-MB-231 cancer cells treated with the compounds at their IC_50_ concentrations.

Compound	Bcl-2 ng/mg protein	Bax ng/mg protein	Bax/Bcl-2 ratio	Caspase-9 nmol/mg protein	Caspase-3 nmol/mg protein
Control (1% DMSO)	12.31 ± 0.97	6.92 ± 0.51	0.56	3.07 ± 0.22	2.56 ± 0.17
**5e**	8.76 ± 0.42^b^	9.05 ± 0.77^a^	1.033	6.98 ± 0.73^c^	6.12 ± 0.54^c^
**10g**	5.90 ± 0.53^c^	12.47 ± 1.08^c^	2.11	8.27 ± 0.139^c^	9.48 ± 0.73^c^

Data are mean ± SD of three separate experiments.

^a^Significantly different from control (1% DMSO) at *p* < 0.05.

^b^Significantly different from control (1% DMSO) at *p* < 0.01.

^c^Significantly different from control (1% DMSO) at *p* < 0.001.

#### Effects on the level of active caspase-9 and caspase-3

The down-regulation of the anti-apoptotic Bcl-2 results in increased levels of free pro-apoptotic Bax which then accumulates at the inner mitochondrial membrane forming channels thus altering membrane permeability. Apoptotic factors then leak into the cytoplasm resulting in the activation of caspases cascade^59^. Caspases are cysteine-containing aspartic acid-specific proteases, which exist as zymogens in the cytoplasm and play a pivotal role in the execution of cell death upon activation^60^. Caspase-3 is the key executioner protease that is activated by upstream initiator caspases such as caspase-9^60^. Therefore, the elevated Bax/Bcl-2 ratios obtained with these treatments triggered the investigation of the protein expression levels of active caspases-9 and -3.

**Figure 4.**
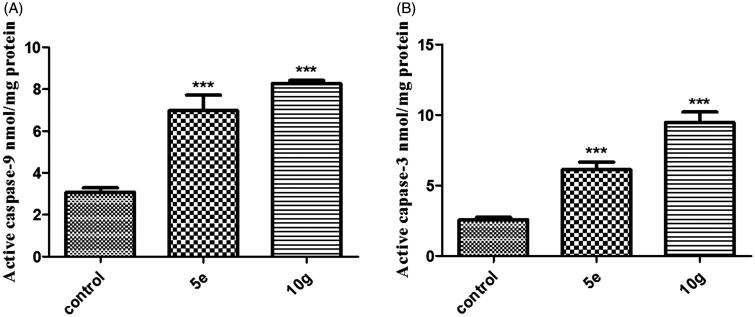
Effect of compounds **5e** and **10g** on the protein levels of A) active caspase-9; B) active caspase-3 in MDA-MB-231 cells treated with the compounds at their IC_50_ concentrations against control (1% DMSO). Data are mean ± SD (*n* = 3). The experiment was done in triplicates. ***Significantly different from control at *p* < 0.001.

In this study, treatment of MDA-MB-231 cells with compounds **5e** and **10g** resulted in a significant elevation of active caspase-9 protein levels by around 2.3 and 2.7 folds, respectively, compared to control (Figure 4(A), Table 2). Moreover, MDA-MB-231 cells upon treatment with compounds **5e** and **10g**, exhibited a significant upregulation of active caspase-3 protein levels by ∼2.4 and 3.7 folds, respectively, compared to control (Figure 4(B), Table 2).

It is worth noting that upon comparing the relative expression levels of caspases with the relative elevation patterns of Bax/Bcl-2 ratio observed in both treatments, it was found that the magnitude of the Bax/Bcl-2 ratio elevation is proportional to that of caspase-9 and that of caspase-3 up-regulation. This observation strengthens the correlation between the increased Bax/Bcl-2 ratio and the activation of proteolytic caspases that act as key players in the execution of apoptosis, which might be responsible for the anti-proliferative activity of the tested compounds. Moreover, compound **10g e**xhibited a more potent activity than compound **5e** in terms of both elevating Bax/Bcl-2 ratio and further activating the caspases cascade at a relatively lower IC_50_. This finding directed further investigation of the apoptotic effect of this compound on MDA-MB-231 cells.

#### Annexin V–FITC apoptosis assay

Externalization of the phospholipid phosphatidylserine (PS) at the cell membrane is one of the hallmarks of cells going into apoptosis^61^. Having a high Ca^2+^-dependent binding affinity to negatively charged phospholipid surfaces. Annexin A5 (AnxV A5), a 36-kDa human protein, is a suitable candidate for apoptosis imaging. Besides, Anx V-based flow cytometry analysis is a useful tool to comprehend whether cell death is due to physiological apoptosis or nonspecific necrosis^61^.

**Figure 5.**
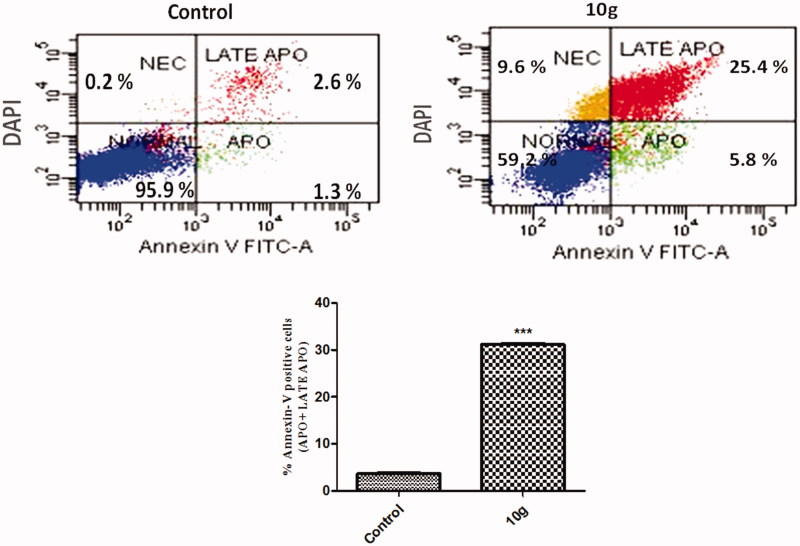
Effect of compounds **10g** on the percentage of Annexin V-FITC-positive staining in MDA-MB-231 cells versus control (1% DMSO). Data are mean ± SD (*n* = 3). The experiments were done in triplicates. The four quadrants identified as: Normal, viable; Apo, early apoptotic; LATE APO, late apoptotic; NEC, necrotic. ***Significantly different from control at *p* < 0.001 (student’s *t*-test).

Further evaluation of the apoptotic effect of the most potent compound **10g** was carried out using Anx V-FITC/DAPI dual staining assay (Figure 5). MDA-MB-231 cells treated with compound **10g** showed a significant increase in the percent of Anx V-FITC positive apoptotic cells (apoptotic and late apoptotic) from 3.88 to 31.21% which comprises about 8.4-folds (*p* < 0.001) compared to control (Figure 5). Flow cytometric analysis of the differential binding of the cells to Anx V-FITC displayed a significant increase in the proportion of early apoptotic cells. This observation is in line with the simultaneous up-regulation of the upstream caspase-9 (an initiator caspase) together with the downstream caspase-3 (the hallmark key executor) belonging to the intrinsic apoptotic pathway. This strongly suggests as well that a cascade of proteinases’ activation has been triggered as a consequence of an increase in Bax/Bcl-2 ratio, eventually leading to apoptosis. These findings clearly imply that compound **10g** has potent pro-apoptotic activity that unravels the mechanism of its anti-proliferative activity.

In conclusion, the reduced expression of the anti-apoptotic protein Bcl-2 in addition to the enhanced expression of the pro-apoptotic protein Bax as well as the up-regulated active caspase-9 and caspase-3 levels together with a harmonized increase in the Bax/Bcl-2 ratio, suggests that the cytotoxic effect of compounds **5e** and **10g** might be attributed, at least in part, to the induction of the intrinsic apoptotic mitochondrial pathway.

#### *In vitro* anti-proliferative activity against four different cell lines

The *in vitro* anti-proliferative activity of the prepared hybrids **5a–h**, **10a–h** and **13a–c** was further examined against a panel of cell lines, namely A549 alveolar carcinoma, Caco-2 colon cancer, LoVo human colorectal carcinoma and HepG2 hepatocellular carcinoma using SRB colorimetric assay as described by Skehan et al.^62^, to carry out further elaboration for their anti-proliferative activity. The results were expressed as IC_50_ values and listed in Table 3.

**Table 3.** *In vitro* anti-proliferative activities of the newly synthesized hybrids against A549, Caco-2, LoVo and HepG2 cell lines.

Compound	IC_50_ (μM)^a^			
	A549	Caco-2	LoVo	HepG2
**5a**	97.04 ± 4.31	NA^b^	15.71 ± 1.52	NA^b^
**5b**	NA^b^	NA^b^	17.58 ± 2.18	NA^b^
**5c**	NA^b^	NA^b^	36.59 ± 3.05	NA^b^
**5d**	28.73 ± 1.59	NA^b^	NA^b^	NA^b^
**5e**	NA^b^	NA^b^	14.48 ± 0.78	NA^b^
**5f**	37.51 ± 2.08	NA^b^	23.77 ± 1.75	NA^b^
**5g**	NA^b^	NA^b^	13.86 ± 0.96	NA^b^
**5h**	NA^b^	NA^b^	39.00 ± 2.13	NA^b^
**10a**	NA^b^	NA^b^	13.83 ± 0.66	NA^b^
**10b**	NA^b^	NA^b^	38.92 ± 2.73	NA^b^
**10c**	NA^b^	NA^b^	15.38 ± 0.83	NA^b^
**10d**	18.42 ± 1.52	NA^b^	25.46 ± 2.07	NA^b^
**10e**	91.57 ± 5.36	NA^b^	14.29 ± 1.32	NA^b^
**10f**	56.71 ± 2.93	NA^b^	13.09 ± 0.33	NA^b^
**10g**	NA^b^	NA^b^	14.40 ± 1.92	NA^b^
**10h**	NA^b^	NA^b^	16.91 ± 1.36	NA^b^
**13a**	NA^b^	NA^b^	48.62 ± 2.21	NA^b^
**13b**	NA^b^	NA^b^	NA^b^	NA^b^
**13c**	NA^b^	NA^b^	NA^b^	NA^b^
Dox.	0.82 ± 0.05	3.01 ± 0.17	5.23 ± 0.49	2.74 ± 0.11

a IC_50_ values are the mean ± SD of three separate experiments.

b NA: Compounds having IC_50_ value >100 μM.

The presented data revealed that some hybrids displayed moderate to fair anti-proliferative activity toward A549 cell line. In particular, compounds **5d** and **10d** showed the better activity against A549 with IC_50_ values of 28.73 ± 1.59 and 18.42 ± 1.52 μM, respectively. Besides, most compounds possessed good to moderate activity against LoVo cell line with IC_50_ values ranged from 13.09 ± 0.33 to 48.62 ± 2.21 μM. Regrettably, among the tested cancer cell lines, the colon Caco-2 and hepatocellular HepG2 were not susceptible cell lines to almost all hybrids influence.

##### ADME study

The ADME of the prepared hybrids **5a–h, 10a–h** and **13a–c** was predicted through a theoretical kinetic study performed by Discovery Studio software (Table 4). To evaluate the lipophilicity and polar surface area, AlogP98 and PSA_2D descriptors were calculated. Besides, absorption, solubility and CYP2D inhibition levels were predicted. All compounds were expected to have very low-to-low aqueous solubility. Meanwhile, most of the examined derivatives seemed to possess good to poor absorption levels, and predicted to be CYP2D inhibitors except compounds **10a**, **10e–h** and **13b** which are expected not to inhibit CYP2D.

**Table 4.** Computer aided ADME study for the prepared hybrids.

Compound	Alog*P*98^a^	PSA_2D^b^	Solubility^c^	Solubility level^d^	Absorption level^e^	CYP2D6^f^	CYP2D6 probability
**5a**	4.911	76.766	−6.987	1	0	1	0.534
**5b**	5.117	76.766	−7.346	1	0	1	0.544
**5c**	5.576	76.766	−7.757	1	1	1	0.613
**5d**	5.660	76.766	−7.834	1	1	1	0.574
**5e**	5.576	76.766	−7.784	1	1	1	0.524
**5f**	5.781	76.766	−8.138	0	1	1	0.683
**5g**	6.240	76.766	−8.549	0	2	1	0.673
**5h**	6.324	76.766	−8.626	0	2	1	0.584
**10a**	4.306	76.766	−6.276	1	0	0	0.455
**10b**	4.511	76.766	−6.638	1	0	1	0.613
**10c**	4.970	76.766	−7.050	1	0	1	0.514
**10d**	5.054	76.766	−7.127	1	0	1	0.514
**10e**	4.970	76.766	−7.035	1	0	0	0.356
**10f**	5.176	76.766	−7.390	1	0	0	0.316
**10g**	5.635	76.766	−7.802	1	1	0	0.386
**10h**	5.719	76.766	−7.879	1	1	0	0.316
**13a**	6.701	67.308	−8.103	0	2	1	0.544
**13b**	7.365	67.308	−8.777	0	3	0	0.366
**13c**	6.906	67.308	−8.316	0	2	1	0.544

a Lipophilicity descriptor.

b Polar surface area.

c Solubility parameter. (0:−2 = optimal, −2:−4 = good, −4:−6 = low, −6:−8 = very low).

d Solubility level (0 = extremely low, 1 = very low but possible, 2 = low, 3 = good, 4 = optimal).

e Absorption level (0 = good, 1 = moderate, 2 = low, 3 = very low).

f CYP2D inhibition (0 = non inhibitor, 1 = inhibitor).

## Conclusion

In an effort to develop potent anti-proliferative agents, the molecular hybridization approach was adopted to design and synthesize new different series of isatin-quinazoline hybrids **5a–h**, isatin-phthalazine hybrids **10a–h** and *N*-benzylisatin-quinazoline hybrids **13a–c**. The *in vitro* anti-proliferative activity of the newly synthesized hybrids was evaluated against the TNBC MDA-MB-231 breast cancer. Compounds **5e** and **10g** displayed the highest potency toward MDA-MB-231 with IC_50_ values of 12.35 ± 0.12 and 12.00 ± 0.13 μM, respectively. Subsequently, compounds **5e** and **10g** were further estimated for their apoptosis induction potential. Both compounds **5e** and **10g** proved to induce apoptosis, which was assured by the reduced expression of the anti-apoptotic protein Bcl-2 in addition to the enhanced expression of the pro-apoptotic protein Bax as well as the up-regulated active caspase-9 and caspase-3 levels together with a harmonized increase in the Bax/Bcl-2 ratio. Furthermore, compound **10g** showed a significant increase in the percent of annexin V-FITC positive apoptotic cells from 3.88 to 31.21% which comprises about 8.4 folds compared to control. Finally the newly prepared hybrids were examined against a panel of cell lines, namely, LoVo human colorectal carcinoma, A549 alveolar carcinoma and Caco colon cancer cell lines to carry out further elaboration for their anti-proliferative activity. Based on the previous findings, we came into conclusion that the isatin- quinazoline/phthalazine hybrids are a good platform for further optimization as novel anticancer agents for TNBC.
